# Supplementary material for: Polyketide synthase gene clusters in the ericoid mycorrhizal fungus Oidiodendron maius: genome-wide prediction, expression profiling, and functional characterization of knockout mutants
Source: Front Plant Sci. 2026 Sep 16;17:1924430. doi: 10.3389/fpls.2026.1924430 (PMC13623903; doi:10.3389/fpls.2026.1924430)
Supplement: Supplementary file 1 [file SupplementaryFile1.zip › Supplementary Materials/Supplementary Folder 1.zip!/Oidma1_AssemblyScaffolds_Repeatmasked/knownclusterblast/cluster55/jgi.p_Oidma1_173659_mibig_hits.html]

| MIBiG Protein | Description | MIBiG Cluster | MiBiG Product | % ID | % Coverage | BLAST Score | E-value |
| --- | --- | --- | --- | --- | --- | --- | --- |
| AGO86660.1 | putative\_fungal\_transcription\_factor | BGC0001255 | Equisetin\_biosynthetic\_gene\_cluster | 42 | 99.4505494505 | 401 | 2e-111 |
| AGN71612.1 | putative\_transcriptional\_regulatory\_protein | BGC0000027 | Azaphilone\_biosynthetic\_gene\_cluster | 39 | 71.2454212454 | 281 | 2.3e-75 |
| AAT65715.1 | putative\_Zn2Cys6\_transcription\_factor | BGC0000629 | Aflatrem\_biosynthetic\_gene\_cluster | 35 | 20.695970696 | 70 | 8.2e-12 |
